# Demonstration of Pressure Wave Observation by Acousto-Optic Sensing Using a Self-Mixing Interferometer

**DOI:** 10.3390/s23073720

**Published:** 2023-04-04

**Authors:** Sébastien Maqueda, Julien Perchoux, Clément Tronche, José Javier Imas González, Marc Genetier, Maylis Lavayssière, Yohan Barbarin

**Affiliations:** 1 Laboratoire d’Analyse et d’Architecture des Systèmes (LAAS-CNRS), Centre National de la Recherche Scientifique (CNRS), Institut National Polytechnique de Toulouse (INPT), Université de Toulouse, 2 Rue Charles Camichel, 31000 Toulouse, France; 2 Commissariat à l’Energie Atomique et aux Energies Alternatives (CEA), Direction des Applications Militaires (DAM), 46500 Gramat, France

**Keywords:** shock waves, self-mixing interferometer, laser sensor, acousto-optic sensor

## Abstract

In this paper, we demonstrate that a compact and inexpensive interferometric sensor based on the self-mixing effect in the laser cavity can be used for the characterization of shock waves. The sensor measures the changes in the refractive index induced by the shock wave. It is based on the self-mixing interferometry scheme. We describe the architecture of the dynamic sensor and the design of the experimental setup used for the characterization that involves a shock tube. Thus, we detail the experimental measurements for shock wave pressure amplitude of 5 bar and address their interpretation with regard to the most admitted models for acousto-optics.

## 1. Introduction

In-depth understanding of the physics at work in blasts generated by energetic materials explosions requires precise and dynamic sensors to characterize the experiments and validate the parameters of the model [1]. Modeling is often based on the Rankine—Hugoniot equations [2], which links the physical properties in the two possible states in the moving front shock wave. Different physical properties interplay: the velocity, the temperature and the overpressure (the pressure above the ambient value). The Friedlander equation [3], with only two parameters (the hydrostatic overpressure and the time of the positive phase), is commonly used to approximate the overpressure of a blast wave as a function of time. For instance, it fits the common TNT (trinitrotoluene) explosive very well, which explains why it is widely used. Energetic materials require more complex modeling to explain phenomena like turbulent combustion. Such modelling takes into account the chemistry of the event with a description of the mixing process inside the fireball [4]. Measurement of the dynamic overpressure is the main measurement of the blast shock wave. Indirectly, it also provides the chronometry (velocity) of the phenomenon since both source and sensor positions and the exact time of the detonation are known. The sensor should be able to measure within 10 to 100 ns an abrupt increase of pressure up to a peak of several bar or even a few tens of bar. Then, the positive pressure decay varies between a few hundreds of microseconds and several milliseconds. Finally, the negative phase is much slower (up to a second). The commercial pressure sensors used for these dynamic measurements usually rely on piezoelectric [5] or piezoresistive technologies; a short review is available in [6]. The main constraint of these sensors is their bandwidth which is limited to several MHz for the fastest of them.

Optical sensors may have many advantages for dynamic pressure measurements in terms of bandwidth, signal communication over a long distance and even immunity to electromagnetic disturbances. Fiber based sensors have been investigated between 2000 and 2006 [7,8]. A small cavity was manufactured at the top of the fiber tip. The latest experiments published showed measurements up to 8 bar. It seems that this approach was not further developed. It could be due to the delicate manufacturing steps required. Cleanroom equipment is designed for planar wafers and not specifically for optical fibers tips. In this paper, another optical approach is proposed. It is based on a Self-Mixing Interferometer (SMI) [9,10]. A laser beam perpendicular to the shock wave propagation and pointing at a distant reflector probes the pressure level in between. The sensor is sensitive to the change in refractive index, that is the actual physical parameter measured. Therefore, a proper physical model is required to get the overpressure as a function of time. It is described in the following section together with the physics underlying the working principle of the Self-Mixing Interferometer. In Section 3, the experimental setup for the shock tube experiment is described. The shock tube generates a pressure step in a controlled way, considered ideal for this first proof of concept. The two models used to transform a number of interference fringes into an overpressure level are discussed in Section 4.

## 2. SMI Sensing of Shock Waves

### 2.1. SMI Principle

The laser cavity, when it is subject to optical feedback from a distant reflector or a scatterer, is the place of wave interferences that results in modifications of the laser emitted power and wavelength. Since Lang and Kobayashi’s early publication [9], the modelling of the self-mixing interferometry is well established through an extension of the laser rate equations that takes into account the backscattered wave re-entering the laser cavity. It has been extensively discussed in previous literature [10,11] that the strong coupling between the phase and the amplitude of the laser electric field induces a complex and non-linear relationship between the round-trip time in the external cavity and the laser emitted power. The resulting interferometric signals that can be observed while monitoring the laser power or its junction voltage share a periodic fringe nature with usual interferometers where each fringe represents a variation of the optical path in the external cavity round-trip of λ/2. The relation between the laser power under feedBack ($P_{\mathrm{FB}}$) and the phase shift of the reinjected light wave $\phi_{\mathrm{FB}}=2\pi \nu_{\mathrm{FB}}\tau_{\mathrm{ext}}$ is written in (1).

(1) $$P_{\mathrm{FB}}=P_{s}\left(1+m\cdot \cos\phi_{\mathrm{FB}}\right),$$

where $\tau_{\mathrm{ext}}$ is the round-trip time of flight in the external cavity, $P_{s}$ is the power emitted by the standalone laser and *m* a modulation index that mostly depends on the ratio of the reinjected power and emitted power $\kappa_{\mathrm{ext}}$ [12,13]. Since the laser frequency $\nu_{\mathrm{FB}}$ (and as a consequence, the phase shift $\phi_{\mathrm{FB}}$) is also affected by the feedback, the fringe shape can vary from almost sinusoidal to very sharp saw-tooth, while side effects such as fringe amplitude hysteresis [14] or fringe disappearance [15] may appear when the cavity’s coupling coefficient *C* reaches large values. This coupling coefficient *C* depends on the ratio between the round-trip time of flight in the external cavity $\tau_{\mathrm{ext}}$ and in the laser inner cavity $\tau_{\mathrm{in}}$, and on the linewidth enhancement factor α as in (2)

(2) $$C=\frac{\tau_{\mathrm{ext}}}{\tau_{\mathrm{in}}}\kappa_{\mathrm{ext}}\sqrt{1+\alpha^{2}}.$$

This expression highlights that the optical feedback coupling increases with the target reflectivity and/or the external cavit length.

While usual applications of SMI are velocimetry or vibration sensing, in a seminal work [16], Bertling et al. have demonstrated the ability of SMI systems to sense the pressure variation in the acoustic domain by using the acousto-optic effect. In this configuration, the laser is beaming onto a target at a fixed distance $L_{\mathrm{ext}}$ and the variation of the refractive index δn induced by the shock wave in the external cavity impacts the phase shift $\phi_{\mathrm{FB}}$ as $\tau_{\mathrm{ext}}$ is decomposed into a constant term $\tau_{0}$ and a time-varying term δτ(t) as in (3)

(3) $$\delta \tau \left(t\right)=\frac{2\cdot L_{\mathrm{ext}}}{c}\delta n\left(t\right).$$

In the context of shock waves in detonic applications, δn is likely to be impacted not only by the pressure burst, but also by abrupt changes in temperature and humidity that could be associated with the explosion. The following section proposes a model of the evolution of the refractive index in detonic conditions.

### 2.2. Shock Waves Acousto-Optics Modeling

In detonic experiments, shock waves cause a strong variation in the refractive index. This section aims to establish a relationship between the variation δn measured by the SMI sensor and the overpressure ΔP induced by the shock wave which can be expressed as

(4) $$\Delta P=P-P_{0},$$

where *P* is the maximum pressure at the front shock and $P_{0}$ the ambient pressure. Schardin [17] applied the Gladstone-Dale empirical law and the Rankine-Hugoniot shock wave theory to calculate δn for shock waves in air. With this approach, the overpressure can be expressed in a literal formula composed of the refractive index variation δn and other known parameters.

The Rankine-Hugoniot theory [18] is used to calculate the overpressure ΔP from the value of the heat capacity ratio γ, the gas density ρ at the shock front, the ambient pressure $P_{0}$ and the ambient density $\rho_{0}$ as:

(5) $$\Delta P=\frac{2\cdot \gamma \cdot P_{0}\left(\frac{\rho }{\rho_{0}}-1\right)}{\gamma +1-\frac{\rho }{\rho_{0}}\left(\gamma -1\right)}.$$

The gas’s specific heat coefficient γ depends on the number of degrees of freedom of the molecules in the gas. In our case, the shocked gas, the ambient air, is considered diatomic. Therefore, γ is a constant equal to 7/5 (=1.4). This assumption is valid up to pressure levels of 20 bar [18]. Beyond this limit, this coefficient will decrease due to the increase in energy, and its variation should be considered.

The ambient density $\rho_{0}$ is given by the ideal-gas equation of state (6)

(6) $$\rho_{0}=\frac{M\cdot P_{0}}{R\cdot T_{0}},$$

where $T_{0}$ is the temperature measured before the shock wave, *R* is the gas constant and *M* is the gas molar mass.

Then, the Gladstone-Dale law [19] relates directly the gas density ρ to the refractive index *n* during the shock

(7) $$\rho =\frac{n-1}{k},$$

where *k* is the Gladstone-Dale coefficient. In the near-infrared light of the laser, *k* we have estimated it to be $0.2296\times 10^{-3}\;m^{3}.{\mathrm{kg}}^{-1}$ in air and at the initial conditions by an extrapolation of Merzkirch data [20].

The refractive index *n* is defined as:

(8) $$n=n_{0}+\delta n,$$

where $n_{0}$ is the refractive index in air at initial conditions, and it is calculated by the Gladstone-Dale law with $\rho =\rho_{0}$ and $n=n_{0}$.

As a result, the combination of expressions (5) to (8) gives the following equation for the overpressure function:

(9) $$\Delta P=\frac{2\cdot \gamma \cdot P_{0}\left(\frac{R\cdot T_{0}\left(n_{0}+\delta n-1\right)}{M\cdot P_{0}\cdot k}-1\right)}{\gamma +1-\frac{R\cdot T_{0}\left(n_{0}+\delta n-1\right)}{M\cdot P_{0}\cdot k}\left(\gamma -1\right)}.$$

According to Rankine-Hugoniot theory, this equation is valid only for rising pressure; relaxation will follow a different behavior. In the case of the shock tube experiment [21], two different pressure steps can be considered, where the pressure rises and can be measured by the SMI system.

### 2.3. Acousto-Optics Modeling Applied to Shock Tube Pressure Measurements

The shock tube is composed of a driver section and a driven section, which are separated by a diaphragm. The driven side is a low-pressure chamber and the purpose of the experiment consist in increasing the pressure level in the driver section until the diaphragm burst. That bursting creates a shock wave that propagates into the driven section. Then, the pressure sensors placed on the driven section observe the pressure levels produced by the shock propagation.

There are two types of pressure measurement on the shock tube. On one hand, the pressure sensors which are placed at the end of the tube measure the reflected pressure; the shock wave arrives perpendicularly to the sensor surface. On the other hand, pressure sensors placed on the side wall of the driven section measure the incident pressure and later the reflected pressure; the shock wave propagates in a direction parallel to the sensor surface and sweeps the active surface. Our optical sensor allows measuring pressure variations caused by the shock wave along the beam between the laser diode and the reflector. Therefore, it is better suited to measure the incident pressure levels.

In this case, the measurement of incident pressure is characterized by two overpressure steps called $P_{2}$ and $P_{5}$ [22]. The first step $P_{2}$ is due to the propagation of the shock wave from the driver section to the driven section. It can be deduced with expression (9) from the previous section:

(10) $$P_{2}=P_{0}+\frac{2\cdot \gamma \cdot P_{0}\left(\frac{R\cdot T_{0}\left(n_{0}+\delta n_{2}-1\right)}{M\cdot P_{0}\cdot k}-1\right)}{\gamma +1-\frac{R\cdot T_{0}\left(n_{0}+\delta n_{2}-1\right)}{M\cdot P_{0}\cdot k}\left(\gamma -1\right)}.$$

Then, the shock wave is reflected on the tube’s end wall and the optical sensor will detect the second pressure step $P_{5}$. The associated expression is not the same as $P_{2}$ because the relation between the pressure $P_{5}$ and the density $\rho_{5}$ becomes:

(11) $$P_{5}=\frac{\rho_{5}}{\rho_{0}}\cdot \frac{T_{5}}{T_{0}}\cdot P_{0},$$

where $\rho_{5}$ is given by the optical sensor:

(12) $$\rho_{5}=\frac{n_{0}+\delta n_{5}-1}{k}.$$

And the ratio $\frac{T_{5}}{T_{0}}$ is expressed as a function of the Mach number $M_{s}$ [22]:

(13) $$\frac{T_{5}}{T_{0}}=\frac{\left[2\left(\gamma -1\right)M_{s}^{2}+3-\gamma \right]\left[\left(3\cdot \gamma -1\right)M_{s}^{2}-2\left(\gamma -1\right)\right]}{{\left(\gamma +1\right)}^{2}M_{s}^{2}},$$

where $M_{s}=\frac{U_{s}}{a_{0}}$ with $U_{s}$ the shock wave velocity which is given by the chronometry between two reference pressure sensors separated by a known distance on the side wall after the diaphragm. $a_{0}$ is the speed of sound in the driven section gas (here ambient air).

Thus, the pressure $P_{5}$ can be expressed with the combination of (11)–(13) as

(14) $$P_{5}=\frac{R\cdot T_{0}\left(n_{0}+\delta n_{5}-1\right)}{M\cdot k}\frac{\left[2\left(\gamma -1\right)\frac{U_{s}^{2}}{a_{0}^{2}}+3-\gamma \right]\left[\left(3\cdot \gamma -1\right)\frac{U_{s}^{2}}{a_{0}^{2}}-2\left(\gamma -1\right)\right]}{{\left(\gamma +1\right)}^{2}\frac{U_{s}^{2}}{a_{0}^{2}}}.$$

Finally, the formulas $P_{2}$ and $P_{5}$ are expressed with known parameters only. It allows linking the overpressure signal ΔP(t) to the variation in refractive index δn(t) measured by the optical sensor. The next sections describe the experimental setup and discusses the results obtained.

## 3. Experiments

### 3.1. Experimental Setup

The setup used for the characterization of the optical feedback interferometer was composed of a metrological shock tube with an inner diameter of 110 mm. An additional chamber was connected at the end of the shock tube to allow optical accesses provided by four fused silica windows, each at 90° around the end of the shock tube driven section. A commercial reference sensor (PCB Piezotronics 113B24) was mounted between two windows to measure the pressure changes within the same location.

**Figure 1:**
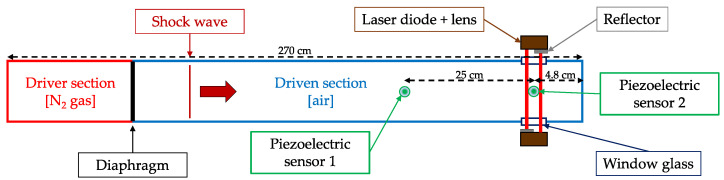
Schematic illustration of optical sensor implementation on the shock tube at a CEA Facility. The two SMI systems are facing each other. Two reference sensors measure the pressure in two different locations.

Two SMI sensing systems were placed outside the shock tube, perpendicularly to the additional chamber at the end of the tube. The lasers were positioned in front of one window, and the target—a flat surface covered with retro-reflective tape—outside the facing window. Each of these optical devices is composed of a single-mode laser diode Thorlabs L1310P5DFB at 1310 nm and a collimating lens. The laser casings were covered with microbeads in order to place them face to face and each casing becomes a target for the other. A schematic of the setup is presented on Figure 1.

Hence, the portion of the external cavity where a change of the refractive index occurs during the shock corresponds to the internal width of the shock tube (110 mm).

The shock tube driver section is filled with nitrogen gas while the driven section is filled with ambient air at atmospheric pressure. The diaphragm which separates the two sections is a standard nickel rupture disc that opens fully and responds within milliseconds to the applied overpressure. On bursting, it creates a shock wave with a rise time shorter than 10 ns that propagates within the driven section until it reflects on the end wall of the tube. The shock wave propagates perpendicular to the laser beam axis, modifying the refractive index of the air in the driven shock tube section, thus changing the apparent optical path sensed by the laser.

The SMI diode output is connected to a conditioning circuit, with a 4 MHz bandwidth for one and 20 MHz for the other.

The output voltage of both the self-mixing interferometer and the pressure sensor were digitized with 14-bit resolution and recorded at a sampling rate of 100 MHz by an HBK GN412 high-speed digitizer in a Gen7t Genesis platform. The experiment duration time was 35 ms.

### 3.2. Results

#### 3.2.1. SMI Signal Processing

As mentioned in Section 2.1, the resulting SMI signal shows fringes with a saw-tooth like shape that are characteristic of a variation in the optical path. The signal processing consists in counting the fringes and observing the orientation of the saw-tooth as this indicates an increase or decrease of the optical path within the external cavity. Thus, the purpose of the signal processing is to determine the change in the fringe number as a function of time while counting them positively or negatively depending on their orientation. Currently, the signal processing is done with an algorithm that automatically counts the fringes. It is being improved and indeed uses empirically adjusted amplitude threshold and Matlab’s *findpeaks* function. In the future, the development of the algorithm is planned, tending as much as possible towards a more robust and automated detection of the interferometric fringes.

Each fringe represents an increase or a decrease of λ/2, the index variation δn can be expressed as:

(15) $$\delta n=\frac{\lambda \cdot N_{\mathrm{fringes}}}{2\cdot L_{\mathrm{ext}}}.$$

The fringe number $N_{\mathrm{fringes}}$ is determined after signal processing while the other parameters $L_{\mathrm{ext}}$ and λ are known. Then, δn is used in Equation (10) to find the pressure $P_{2}$ and in the Equation (14) for $P_{5}$.

#### 3.2.2. Results

**Figure 2:**
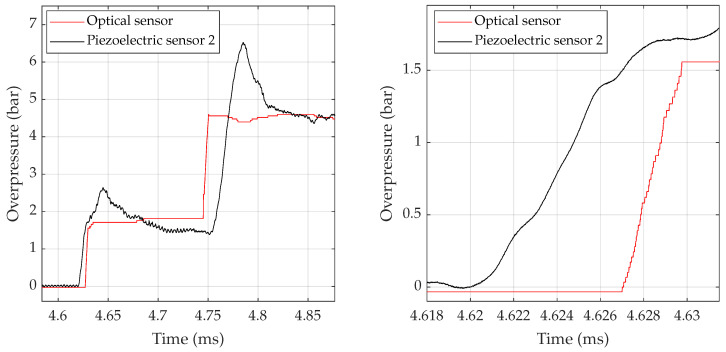
Comparison between an SMI sensor computed pressure change (red solid line) and the piezoelectric sensor 2 (black solid line). Zoom on the steps of pressure $P_{2}$ and $P_{5}$ (**left** plot). Close-up of the first rise (**right** plot).

Figure 2 shows the result of the pressure change as computed from the SMI sensor signal and the piezoelectric sensor 2 for one of the tests performed on the shock tube near 5 bar. An important point is that a faster rise time is observed for the optical sensor as compared to the two piezoelectric sensors. The 2nd piezoelectric sensor (black line) has a 10–90% rise time about 6.7 μs for the first rise and about 17.5 μs for the second rise. While the optical sensor has a rise time about 2.7 μs for the first and 5 μs for the second.

**Figure 3:**
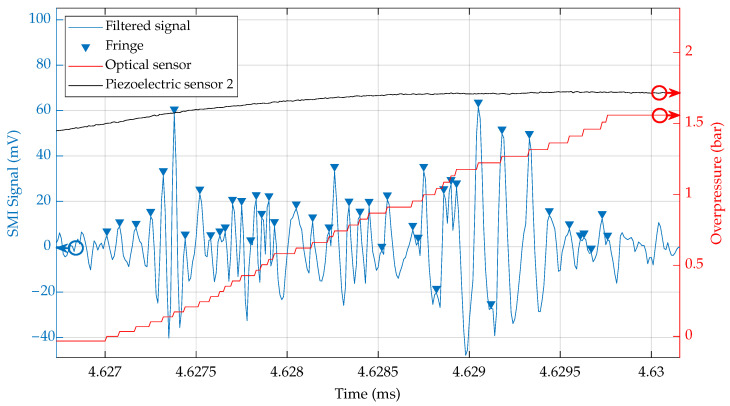
Zoom on the rise of the pressure step $P_{2}$. Highlighting of fringe detections (blue triangles) for the reconstruction of the pressure signal (blue). The reference signal of the piezoelectric sensor 2 (black solid line) and the reconstructed pressure change (red solid line) are also displayed.

In Figure 3, the close-up on the rise of the pressure step $P_{2}$ is plotted for both pressure change sensors (red solid line for the SMI sensor and black solid line for the piezoelectric sensor) as well as the SMI sensor signal after bandpass filtering (blue solid line) with highlighting of the detected fringes that are taken into account for the reconstruction of the pressure change.

**Figure 4:**
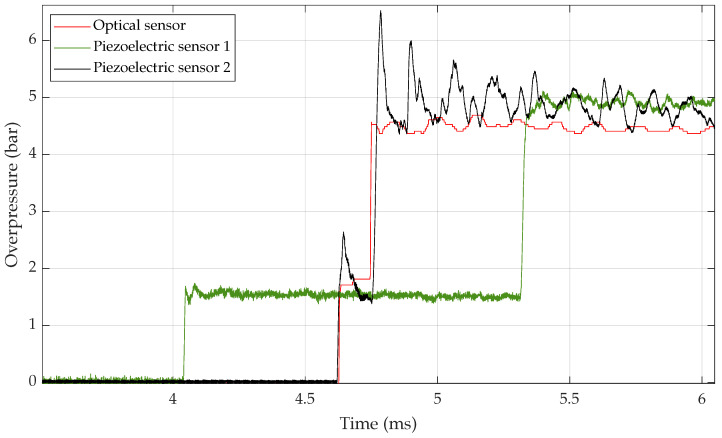
Zoom on the two steps of pressure $P_{2}$ and $P_{5}$. Comparison between the reference piezoelectric sensors and the 20 MHz SMI sensor after signal processing.

Figure 4 shows the response with time of the SMI sensor with bandwidth 20 MHz (red solid line), the piezoelectric sensor 2 (black solid line) located approximately in the same plane as the reference and the piezoelectric sensor 1 (green line) located about 25 cm before the other sensors (in the driven section). The reference piezoelectric sensors indicate a first overpressure step $P_{2}$ around 1.6 bar and a second overpressure step $P_{5}$ of about 5.4 bar. We can also see oscillations in phase opposition on both sensors that are in the same plane. This aspect will be discussed in the next section.

## 4. Discussions

The emphasis of the fringe detection in Figure 3 shows that with the presence of noise and with the complexity of the sensor signal, the counting is not perfect. There are artifacts with missing fringes and signal peaks that should not have been counted, but the high frequency of fringes expected by the model for the pressure rise were present when the shock wave crossed the laser beam.

The Figure 4 shows a consistency between the SMI sensor and both piezoelectric sensors. The pressure levels for the two steps are almost the same with a difference of less than 10%.

The temporality is also consistent. As the shock wave propagates in the driven section, it first passes through piezoelectric sensor 1 and then the piezoelectric sensor 2 before it reflects on the end wall and then passes both sensors in the reverse order. The optical sensor was in the same plane as piezoelectric sensor 2 and their timings match. However, it’s important to notice that the SMI sensor has a better rise time than the piezoelectric sensor. This highlights the higher bandwidth of this optical sensor using the SMI principle.

In Figure 4, there are oscillations around 6.7 kHz, that can be seen with various amplitudes for all the sensors on the overpressure steps. Regarding electrical sensor 2, part of these oscillations is due to the resonance frequency excitation of the mechanical membrane. The sharp peaks observed are a good indication of this. However, since the optical sensors also measure small overpressure variations, these can be attributed to the disturbances created by the flat optical windows mounted in a circular tube [23]. The geometry of the tube part inserted to allow this SMI measurement within the shock tube is far from ideal and can cause interference modes degrading the shock wave planar front. These perturbations might even trigger the resonance effect observed with sensor 2 because sensor 1 is much less perturbed. It was expected for the first step $P_{2}$ because the sensor is located in the main tube. For the second step $P_{5}$, these perturbations might have time to be averaged and damped before again reaching sensor 1.

These experiments using a shock tube have proven the feasibility of measuring high-pressure blasts with response times compatible with those needed in detonation experiments. However, as discussed previously, some discrepancies between the SMI sensor and the piezoelectric reference remain and need to be studied more deeply in connection with the hypothesis made in the modeling.

First, the shock wave was taken to be perfectly planar and then the SMI’s external cavity was modified. In reality, the shock tube used in these experiments is far from ideal: the shock wave generated is known to be curved due to the insufficient length of the driven section, and the beam of the laser diode is not affected instantaneously along its full length. The curvature increases the interaction time between the laser and shock wave. Furthermore, it creates an inhomogeneous index, that can be problematic for systems with long external cavities because the optical sensor measures a mean value of the changing refractive index along the beam.

Second, in order to have a wave with a perfectly flat shock front, a properly dimensioned shock tube must be long enough in relation to its diameter. In this paper, the shock tube used during these experiments did not have the necessary dimensions to generate pressure steps without deformations of the shock front. In the experiments with this shock tube, there are other non-ideal behaviors that are not considered in our study [24]. As a consequence, it is irrelevant to compare performances of our sensors with measurements made in another shock tube. Future experiments will be done on a new, better dimensioned shock tube, that will overcome the other intrinsic defects of the previous shock tube.

Then, the acousto-optic modeling proposed in this paper assumes that the gas in the chamber is composed only of oxygen and nitrogen. In fact, the real mixture is also composed of other gases such as carbon dioxide that can affect the γ coefficient. The humidity may also affect the refractive index and hasn’t been taken into account. The modeling is made for an ideal gas with ideal heat capacities and this assumption is valid for pressure levels up to 20 bar. More complete models of gases exist that would allow them to reach higher pressure, but their use in our acousto-optic model will be more complicated.

Next, the *k*-coefficient of the Gladstone-Dale law used in this paper is estimated from empirical values found in the literature [20]. The variation as a function of the wavelength seems weak but it would be interesting to also verify this value experimentally.

**Figure 5:**
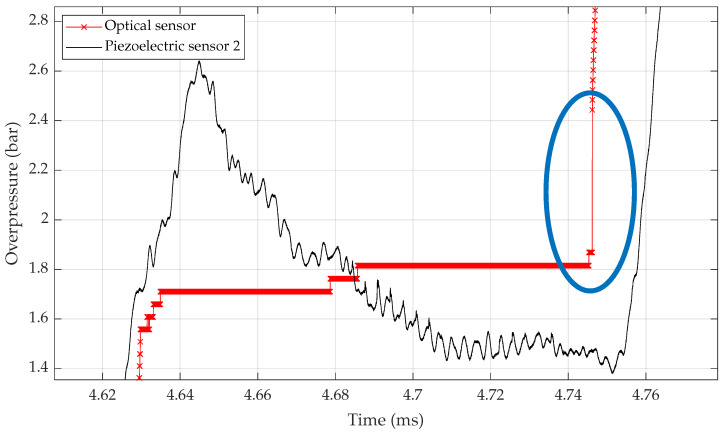
Discontinuity between overpressure steps $P_{2}$ and $P_{5}$ that is created by the use of a distinct acousto-optic models for the two-pressure step. The blue circled area is where points are “missing”.

In the shock tube configuration presented here with two pressure steps, if we want to use both values to calibrate sensors, this last point can be improved. In Figure 5 below, one can see circled in blue a discontinuity between both overpressure steps $P_{2}$ and $P_{5}$. This is due to the use of the two different equations as explained in Section 2.3. For the moment, it isn’t possible for them to have a continuous signal reconstruction with the current model.

The pressure resolution is obtained for an index variation $\delta n_{\min}$ corresponding to a fringe, so it can be expressed as follows:

(16) $$\delta n_{\min}=\frac{\lambda }{2\cdot L_{\mathrm{ext}}}.$$

If we inject this value into the calculation of the overpressure of the first step, we obtain a resolution of 34 mbar. According to the datasheet of the PCB 113B24 sensor, the resolution of the reference sensor was 0.35 mbar, i.e., 100 times less than the optical sensor. It is possible to improve this resolution either by decreasing the wavelength (but there will be laser safety issues), either by increasing the external cavity which will induce possible losses in signal-to-noise ratio as well as eventual disappearance of fringes [15]. We can then deduce an optical sensor sensitivity of about 29 fringes/bar for the first step of our configuration, for the wavelength and external cavity length used in this paper.

## 5. Conclusions

In this paper, we have demonstrated the principle of a sensor to characterize shock waves based on the self-mixing interferometry effect. The proposed system can measure overpressure profiles similar to those present in detonation experiments. The sensor rapidly measures the refractive index changes along a laser beam. To get the overpressure values from this sensor, we proposed a complete acousto-optic model. A few demonstrations were done in a shock tube with overpressure steps of 1.7 and 5.0 bar. After signal processing, very consistent pressure levels were measured. The timing of the phenomena involved is also correct. These tests allowed validating a first version of our acousto-optic model. In the context of the incident pressure measurement, a faster rise time than with commercial sensors was demonstrated. The results also showed the presence of unwanted oscillations that are not attributed to the sensor itself and could be avoided by optimizing the design of the shock tube extension in order to limit pressure oscillations. Further development of this study includes improvement of the electronics of the sensor with in particular a larger bandwidth, as the larger bandwidth sensor (20 MHz) has shown slightly more accurate results as compared to the lower bandwidth one (4 MHz). Moreover, for shocks with higher pressure steps, it is expected the sensor bandwidth to have a strong impact on the pressure change reconstruction. The developments will also focus on the extension of the model for compatibility with on-field experiments and deployment of a shock tube with a better overpressure profile to calibrate all our sensors. This latter part is in progress at the C.E.A. Gramat laboratory.

## Data Availability

Not applicable.

## Abbreviations

The following abbreviations are used in this manuscript:

SMI	Self-Mixing Interferometry
PFB	Power under FeedBack
